# Draft Genome Sequence of “*Candidatus* Bacteroides periocalifornicus,” a New Member of the *Bacteriodetes* Phylum Found within the Oral Microbiome of Periodontitis Patients

**DOI:** 10.1128/genomeA.01485-15

**Published:** 2015-12-23

**Authors:** Jeffrey S. McLean, Quanhui Liu, John Thompson, Anna Edlund, Scott Kelley

**Affiliations:** a Department of Periodontics, University of Washington, Seattle, Washington, USA; b Department of Biology, San Diego State University, San Diego, California, USA; c Microbial and Environmental Genomics, J. Craig Venter Institute, San Diego, California, USA; d School of Dentistry, University of California Los Angeles, Los Angeles, California, USA

## Abstract

Here we present the draft genome of a distantly related member within the phylum *Bacteriodetes*, “*Candidatus* Bacteroides *periocalifornicus*.” The draft genome sequence was assembled with metagenomic data from a patient with periodontitis. The closest relative has less than 68% average nucleic identity, supporting a novel family within *Bacteriodetes*.

## GENOME ANNOUNCEMENT

Periodontal disease is a chronic progressive disease that affects an estimated 5% to 20% of the world's population (1) and has been identified as risk factor for heart disease (2, 3). Periodontal biofilms include numerous pathogens and become increasingly complex at the disease worsens (4). Although periodontal biofilms occur in one of the most heavily investigated microbial ecosystems, the precise relationship between the periodontal biofilm microbes and the severity of the disease is still a hot topic of research. Furthermore, a large percentage of the microbes associated with the oral cavity and periodontal disease remain uncharacterized (5–7). Here, we report the identification and phylogenetic placement of a draft microbial genome assembled from shotgun sequencing data derived from a patient with severe periodontitis.

Sample collection and DNA isolation were performed as described previously (7). Briefly, following the clinical examination, microbial samples were collected from the two deepest periodontal pockets of the dentition using a periodontal scaler. DNA was extracted with the NucleoSpin tissue nucleic acid and protein purification kit (Macherey-Nagel GmbH & Co, Germany). A total of 24 paired-end Illumina libraries from 12 subjects before and after treatment were sequenced on a HiSeq 2000 sequencer (Illumina Inc.) (2 × 100 bp).

All quality-trimmed reads were *de novo* assembled using SPAdes v 2.40 (8, 9). Contigs from each library were taxonomically classified based on a machine learning algorithm using MG Taxa (http://mgtaxa.jcvi.org/) as described previously (10). Several large scaffolds in a number of libraries were originally classified at a low score to uncultivated phylum OD1, indicating they were distantly related to any previously sequenced genome. Scaffolds representing the novel genome were then extracted from a single library and inspected for kmer frequency consistency and used for further analyses.

The draft genome is 2.53 Mb with an overall GC content of 59.4%. Gene annotation using the Prokaryotic Genome Automatic Annotation Pipeline (PGAAP) provided by the National Center for Biotechnology Information (NCBI) identified a total of 1,875 genes, consisting of 1,678 coding sequences, 39 tRNAs, and 1 rRNA operon (5S). A set of 31 single-copy genes were extracted and aligned to 78 sequenced genomes using AMPHORA2 (11). The resulting likelihood-based tree was built from 31 concatenated genes and indicated relatedness to the *Bacteriodetes* phylum; however, the closest genome was *Alistipes putredinis*, with an average nucleotide identity of 68% (12). These results support that the genome represents a novel family member within the *Bacteriodetes* phylum, and the name “*Candidatus* Bacteroides periocalifornicus” is proposed.

### Nucleotide sequence accession numbers.

This whole-genome shotgun project has been deposited at DDBJ/EMBL/GenBank under the accession number LIIK00000000. The version described in this paper is version LIIK01000000.
